# Supplementary material for: Are carbon emissions trading and green financial instruments synergistic? -Comprehensive quantitative research based on content analysis
Source: PLoS One. 2024 Mar 7;19(3):e0298601. doi: 10.1371/journal.pone.0298601 (PMC10919601; doi:10.1371/journal.pone.0298601)
Supplement: S1 Data — (ZIP) [file pone.0298601.s001.zip › Data/地级市Z_F工作报告.zip!/╝¬┴╓/░╫│╟╩╨/└┤╫╘╣1⁄2╓┌║┼í╛┬φ┐╦╩2╛▌═°í┐_2015.docx]

各位代表：

　　现在，我代表市政府，向大会作政府工作报告，请予审议，并请市政协各位委员提出意见。

　　一、2014年工作回顾

　　过去的一年，面对经济下行压力不断加大的严峻形势和改革发展稳定的繁重任务，在省委、省政府和市委的正确领导下，在市人大、市政协的监督支持下，我们紧紧依靠全市人民，开拓进取，攻坚克难，推动白城振兴发展迈出了新的步伐。预计2014年，全市地区生产总值完成750亿元，增长8%。固定资产投资完成610亿元，增长20%。规模以上工业增加值完成222亿元，增长9%。全口径财政收入完成63.8亿元，增长15%；地方级财政收入完成41.4亿元，增长12%。社会消费品零售总额完成284亿元，增长13%。城镇居民人均可支配收入24900元，农民人均纯收入7300元，分别增长8.5%和8.3%。主要经济指标增幅保持全省前列，其中地区生产总值、规模以上工业增加值、全口径财政收入增幅位居全省第一。

　　——经济运行稳中有进。全市新开工重点项目264个，亿元以上项目达到134个。规模以上工业总产值完成648亿元，增长10.2%；工业用电量增长18.6%，全省第一。粮食产量达到82.07亿斤，创历史最高水平。加强“一线八点”旅游线路和生态旅游品牌建设，旅游业收入增长25%，查干浩特旅游开发区、嫩江湾湿地公园晋升为国家4A级景区。加快发展物流、电商、信息等现代服务业，通榆成为阿里巴巴村淘项目全国第三个试点县，洮北电商产业园区已入驻企业62户。服务业增加值完成268亿元，增长7.8%。进出口贸易总额增长5.9%。民营经济主营业务收入1087.6亿元，增长11%。

　　——改革开放全面深化。按照市委部署，围绕建设吉林西部生态经济区，推进综合配套改革，由政府承担的98项重点改革有序开展。落实简政放权，减少行政审批86项，减少行政事业性收费9项。完成卫生、计生机构合并和工商、质监、食药监部门管理体制改革，对176个事业单位进行分类。改革工商登记制度，个体工商户、私营企业户数分别增长10.1%和19%。启动农村金融综合改革试验区，我市第一家地方性银行白城农商行挂牌运营，组建惠农担保公司，新增小贷公司31家。推进电力市场化交易、土地确权、“粮食银行”、公立医院等改革试点。促进开发区转型升级。加强国家外贸出口转型示范基地建设。全市招商引资到位资金810亿元，增长20%。

　　——基础设施加快建设。新建高速公路42公里，新改建国省干道96.7公里、农村公路571.4公里。推进白阿、长白等重点铁路扩能改造，加快机场建设，开工建设长庆街公铁立交桥。实施分洪入向除险加固、洮儿河堤防治理、引嫩入白收尾配套、月亮泡6号坝防洪等水利工程。通榆500千伏输电线路全面开工。加强城市地下管网改造、街路硬化等设施建设，启动二次供水改造。促进林海镇等特色示范镇建设。“富民强村固基”工程建设项目1669个，改造提升美丽乡村41个。

　　——生态环境持续改善。深入开展“三年再造百万林，建设西部大屏障”活动，造林41万亩；落实草原生态补奖面积853万亩，综合治理草原30万亩，林草覆盖率达到39%。推进河湖连通工程，新连水库泡塘29个，增加蓄水量5.6亿立方米，改善和恢复湿地22.5万亩。恢复建设芦苇湿地20万亩。实施土地整治项目7个，治理盐碱地近7万亩。节能减排任务全面完成。实施大气污染综合整治，空气质量优良天数占93.7%。全年降水量达到473毫米，全市地下水位平均上升1.02米，生态效益逐步显现。我市被确定为全国水生态文明建设试点城市。

　　——民生保障得到强化。全面完成十个方面27件民生实事。城镇新增就业4.8万人，城镇登记失业率3.7%。农村劳动力转移就业38.9万人（次）。实施大学生就业促进计划，就业率达到81.8%。社会保障一卡通列入国家试点。城镇职工基本医疗保险等5个险种实现市级统筹，城镇居民医疗保险报销封顶线由6万元提高到16万元，城镇居民大病医疗保险最高支付限额达到30万元，新农合个人补助标准提高60元，城镇低保月人均提高30元，农村低保年人均提高200元。落实《社会救助暂行办法》，推行救助申报一站式服务。实行70周岁以上老人免费乘坐公交车。建成廉租房3124套，改造农村危房13818户。实施“气化白城”工程，铺设燃气管网82.7公里。推进扶贫攻坚，实现8万农村贫困人口脱贫。解决4万农村人口饮水安全。

　　——社会事业协调发展。申报省级以上科技计划项目79项，4项获省科技进步奖，1项获省自然科学一等奖。洮南敖东、白城福佳分别获得省政府“双十”重大科技成果转化和攻关项目。完成实用技术对接35项。新改建公办幼儿园5所，建设农村标准化学校7所，改造农村薄弱校74所，组建白城职业教育集团。建设标准化村卫生室255所，实现基本药物制度全覆盖。加强传染病防治工作。人口出生率、自然增长率完成省下达的控制目标。落实单独两孩政策。开展“白城好人、引领风尚”活动。大安获得“全国敬老模范单位”荣誉。创编“吉剧振兴”重点剧目《良子》。我市取得省第十七届运动会田径项目金牌榜第二名。全民健身中心正式投入使用。公共机构节能工作深入推进。住房公积金归集率、贷款率全省第一。国家土地例行督察整改工作全面落实。第九次社区居委会换届选举圆满完成。人才、双拥、民族宗教等工作取得新成效，广播电视、妇女儿童、外事侨务、残疾人、老龄、气象、人防、档案、地方志和红十字会等事业进一步发展。

　　——社会大局保持稳定。开展大安全建设，安全生产形势保持平稳，工矿商贸、道路交通和火灾“三类事故”发生起数下降43.4%，获得全省安全生产目标责任制考核一等奖。推进“平安白城”建设，加强城乡巡逻防控，开展反恐防暴演练，刑事、治安案件分别下降3.6%和8.8%。深入实施“六五”普法规划。进一步加强行政复议。加大信访维稳力度，信访总量下降39%。全年保持无重大食品安全事故、无药品安全事件“双无”目标。强化应急管理，公共突发事件得到有效预防和处置。

　　——自身建设不断加强。深入开展党的群众路线教育实践活动，大力整治“四风”问题。认真办理人大代表建议46件和政协提案112件、建议案2件。全面落实市人大、市政协视察调研活动中提出的28个方面意见和建议。年中分别召开人大代表、政协委员座谈会，听取对政府工作的意见和建议。进一步完善了经济发展、城市建设、社会治理3个联席会议制度。举办科学行政讲堂12期。深化政务公开，开展行政审批“大提速”活动。启动政府部门权力清单工作。强化审计监督作用。加强编制管理，实现机构编制总量“零增长”。“三公”经费下降10.7%。严格落实党风廉政建设责任制，反腐倡廉工作进一步加强。

　　一年来，我们统筹全局抓推进、紧盯重点求突破，着力抓了以下九项工作：

　　（一）建设“铁公机”。投资190亿元、设计时速160公里的长白铁路扩能改造项目开工；白阿铁路扩能改造全线征地，镇西站改造工程开工，新开河铁路桥建成通车；平齐、通让铁路电气化改造工程可研报告通过专家评审。嫩丹高速公路白城至镇赉段竣工通车，镇赉至坦途段、白城至洮南段、白城西联络线、机场连接线开工建设。白城机场全面开工，航站楼暖封闭，航管楼主体封顶，场道工程完成基础，供油、供水等附属工程同步推进。

　　（二）推动“五业振兴”。清洁能源产业：风电、光电新增并网30万千瓦，落实高载能政策，拉动用电量增加5.36亿度，启动大安清洁供暖项目，开展“柴改电”、光伏提水电灌农业试点。农产品加工业：投资4.5亿元的中绿集团粗杂粮加工项目完成厂房主体工程，金塔集团投资2.8亿元新上豆瓣酱、辣椒籽油项目，宏达食品投资1.1亿元新上甜玉米罐头生产线。有6户企业5类产品获国家生态原产地产品保护认证，辣椒、杂粮杂豆质量安全示范区成为国家级示范区。汽配产业：金事达电气与美国安费诺集团、中一精锻与中国兵器工业集团实现战略重组，恒维年产35万辆汽车绞链项目竣工投产，德尔福派克产值达到45亿元。装备制造业：“三一”风电等4户企业恢复生产，成飞叶片产值增长66%，成来变压器完成扩能改造。纺织服装业：四季盛宝启动异地改造，恒盛毛纺完成技改，鑫源棉业百万米织布项目投产，铭威服装工业园产值达到10亿元。全市“五大产业”实现总产值540亿元，占工业总产值83%。

　　（三）推进“六畜兴旺”。坚持规划引领，聘请农业部专家，编制白城市现代畜牧业发展规划，制定实施“六畜兴旺”方案。坚持龙头拉动，引进正邦600万只肉羊、飞鹤30万只奶山羊、金苜神万头肉牛等项目。雏鹰400万头生猪项目建成猪舍461栋、建设70万吨饲料厂。英联30万吨饲料项目投产。坚持质量提升，启动建设无规定疫病区，开工建设白城市农产品质量安全检测中心，实施“精品肉”工程。畜禽饲养量达到4713万头（只），实现牧业产值121亿元。

　　（四）加快生态新区建设。推进路网工程，新改建道路15条、23公里，铺设管网120公里，核心区“七通一平”基本完成。开发鹤鸣养殖场，完成土方140万立方米，建设桥梁9座，56万平方米的湖区实现蓄水。建设绿地系统，绿化主干路5条，栽植乔木3.6万株、绿篱地被10.9万平方米。加强产城融合，碧桂园一期项目竣工开放，市民服务中心主体完工，科文中心、九年制学校、中绿闽台商贸港、凯伦威尼斯水上乐园项目开工建设。

　　（五）实施保基本兜底线工程。实施“水净、城洁、灯亮、路平、房暖、居安、物管，救助特困家庭老人、孩子、残疾人”10项工程。市区改造二次供水管网53.8公里、泵站39座，15万居民实现24小时供水；综合整治老旧小区45万平方米，拆除违章建筑13.4万平方米；改造路灯4237盏，铺设电缆19公里；维修改造道路7条、1.5万平方米，硬化小区10.6万平方米；完成“暖房子”工程90万平方米，改造供热管网20公里，扩大电厂供热面积252万平方米；改造城市危房2691套15万平方米；开展“宜居小区”创建达标活动，加强物业考核管理，改造小区地沟管线50.7公里。采取低保专项、政府投入、公益捐助等措施，对111名特困家庭高龄失能老人、15名患有白血病和先天性心脏病特困家庭儿童、198户特困残疾人家庭进行救助。

　　（六）实施新城家园棚改工程。抢抓新一轮棚改政策机遇，争取开行贷款33亿元。按照“政府主导、分区实施、异地安置、以人为本”的原则，采取“先建后拆”模式，在生态新区开发建设新城家园棚改项目，已开工建设回迁安置住房137栋、97万平方米，连片征拆铁南、铁东、官银号等棚户区。致力于打造精品工程。按照商品房标准设计，全部实行简装修，同步建设道路、供热、供水、燃气等市政配套设施，以及学校、医院、超市、休闲等公共服务设施。致力于打造安全工程。严格建设程序，强化施工质量监管，确保资金规范、工程安全。致力于打造民生工程。实施回迁特困家庭基本生活救助，保障“零就业”家庭基本就业。省政府高度肯定我市棚户区改造模式，并向全省推广。

　　（七）组织岭下镇抗洪救灾。去年7月8日凌晨，查干浩特岭下镇遭受特大洪灾。在省委、省政府和市委的坚强领导下，我们超前部署，及时预警，紧急行动，科学应对，组织部队官兵、公安干警和干部群众1900余人，投入紧急抗洪抢险，抢救老弱病残孕幼900余人，转移群众1.5万人，集中安置3328人、分散安置6672人，确保了无一人伤亡、无疫情发生。及时组织开展生产自救、灾后重建，实行领导包保、部门帮扶、属地组织推动，投资1.5亿元，新建住房1535户、维修住房1159户，修复水毁工程21处、道路18.6公里，灾区人民生产生活得到及时恢复、有效保障。

　　（八）加强信访工作制度化建设。针对接访下访工作，建立完善《信访接待制度》、《信访接待实施办法》、《领导干部接访下访工作实施意见》等制度规定，规范工作程序，明确首接责任、办结时限。针对案件化解工作，建立完善《信访信息排查及报告工作实施办法》、《疑难信访事项化解工作实施办法》等制度规定，明确责任主体、具体职责和工作要求。针对督办落实工作，建立完善《信访督查督办工作规则》等制度规定，实行领导约谈、责任追究倒逼机制，切实解决好群众诉求问题。全年新发生信访事项化解率达到95%以上，信访积案化解率达到82.4%。

　　（九）开展党的群众路线教育实践活动。按照中央、省委和市委的安排部署，市政府党组及各部门严格落实“照镜子、正衣冠、洗洗澡、治治病”的总要求，认真组织开展了为期8个月的教育实践活动。突出学习教育，市政府党组集中学习传达习近平总书记系列重要讲话和中央、省委、市委重要会议精神19次，各部门组织集中学习1400余场次、专题研讨420余场次；坚持问题导向，市政府按照“当前问题马上办、近期问题快推进、长远问题有规划”的原则，重点解决六大类77个突出问题，部门重点解决962个突出问题，深入开展专项整治活动；强化建章立制，市政府建立健全制度规定44项，部门建立健全制度规定940项。通过教育实践活动，党员干部思想受到深刻洗礼，理想信念、宗旨意识普遍增强；“四风”问题得到有力整治，改进作风、革除积弊的行动更加自觉；群众切身利益问题有效解决，党群干群关系进一步密切；普遍经历了一次严格的党内政治生活锻炼，党内政治生活更加严肃规范。

　　各位代表！

　　过去一年的成绩来之不易。这是省委、省政府高度重视、亲切关怀的结果，是市委科学决策、正确领导的结果，是市人大、市政协有效监督、大力支持的结果，是全市上下咬紧牙关、奋力拼搏的结果。在这里，我代表白城市人民政府，向全市各族人民，各民主党派、工商联、人民团体、无党派人士、离退休老同志，向驻白的中省直部门和企事业单位、人民解放军指战员、武警部队官兵、公安司法干警，向关心和支持白城发展的各界朋友，致以崇高的敬意和衷心的感谢！

　　同时，我们也清醒地认识到，全市经济社会发展和政府工作中还存在许多困难和问题，主要表现在：一是经济下行压力继续加大，投资拉动减弱，消费支撑趋缓，出口竞争激烈，企业面临市场疲软和成本上升双重压力，稳增长的任务十分繁重；老工业基地一些深层次的体制机制矛盾在转型升级中愈加突显，产业结构不优、市场化程度不高、创新能力不足，培育新产业、新业态的内生动力亟待增强，白城的许多资源和潜力还没有很好地转化为发展优势。二是改革动力需进一步增强，一些地方和部门体现改革精神的实践创新不足；关联改革的统筹衔接不够，增强改革实效性需下更大功夫；激活要素活力，完善市场机制，发挥市场配置资源决定性作用还需大力探索。三是城市基础设施建设滞后，城市管理体制不顺，改造完善城市功能，提升民生保障水平面临的困难比较大。生态修复的效益正逐步显现，但保护生态和治理环境的挑战依然严峻、任务依然繁重。四是创新社会治理面临许多新情况新问题，化解社会矛盾难度加大，不稳定因素仍然较多，安全生产形势不容乐观，许多涉及群众切身利益的民生问题解决得不够及时、不够彻底。五是政府工作运行的效能需进一步加强，决策、落实、督查机制尚需进一步完善，一些干部适应发展新常态的意识和能力不足，少数单位软弱涣散、个别干部作风不正仍然存在，作风整治的力度不能减弱，廉政建设的高压态势还需继续保持。对于这些困难和问题，无论多么艰难和复杂，我们都要咬住不放，努力攻坚破解。

　　二、2015年工作安排

　　2015年，是全面深化改革、全面推进依法治国的关键之年，也是完成“十二五”规划的收官之年。做好今年的工作，意义重大。省委十届四次全会和全省经济工作会议对适应新常态，推动吉林新一轮振兴发展作出了全面部署。市委五届九次全会分析形势、科学研判、全面部署，明确了今年白城经济社会发展的主要任务。

　　加快吉林西部生态经济区建设，是省委、省政府着眼国家区域发展和对外开放格局作出的重大决策，是吉林省适应经济发展新常态、打造振兴发展新引擎的重大举措，是白城发挥后发优势、实现跨越赶超、完成扶贫攻坚任务的重大机遇。我们要在省委、省政府和市委的坚强领导下，牢牢扭住这一关乎全局的重大部署，奋力开启建设吉林西部生态经济区新的征程。要坚持抓规划引领。通过高起点、高标准、高水平编制好“十三五”规划和各专项规划，突出加快建设吉林西部生态经济区这个核心，体现新常态，落实大战略，融入大格局，明确大目标，谋划大工程，以宏伟的蓝图振奋精神，以科学的规划引领发展。要坚持抓项目带动。把抓项目作为推动发展的“生命线”，作为检验干部的“主战场”，贯穿各领域、各环节。用项目最大程度地展现本地优势，用项目最大范围地整合市场资源，用项目最大限度地聚集政策支撑。在项目的谋划、洽谈、建设中考察和检验干部的思想水平、政策水平、工作能力和作风素质。要坚持抓改革攻坚。当前已经到了必须依靠深化改革才能实现持续健康发展的关键时期。面对现实的困难和矛盾，必须转变思维惯性，突破路径依赖，敢于啃硬骨头、涉险滩，破解制约发展的瓶颈，激发市场和社会活力，促进改革红利转化为白城振兴发展新动能。要坚持抓政策支撑。立足资源禀赋，着眼区域大格局，围绕生态、农业、扶贫和老工业基地振兴四大主题，谋划白城发展大概念，打造上争外引的强磁场、大洼地，以政策集聚叠加构建白城加快发展的新动力。

　　2015年，政府工作总的要求是：深入贯彻党的十八届三中、四中全会和中央经济工作会议精神，按照省委十届四次全会、省经济工作会议和市委五届九次全会的部署，牢牢抓住国家新一轮振兴东北老工业基地的重要机遇，紧紧围绕加快建设吉林西部生态经济区的重大任务，大力实施“三大战略”，全面落实“五大发展”，着力稳增长、转方式、调结构、促改革、惠民生、强法治，努力推动白城新一轮振兴发展。

　　全市经济社会发展主要预期目标是：地区生产总值增长7.5%左右；地方级财政收入增长7%左右；固定资产投资增长15%左右；规模以上工业增加值增长8%左右；社会消费品零售总额增长12%左右；城乡居民人均可支配收入均增长8%左右；城镇新增就业4.2万人，城镇登记失业率控制在4.5%以内；单位GDP能耗、二氧化碳排放强度等节能减排指标完成省下达任务。

　　重点抓好以下十个方面工作：

　　（一）突出项目攻坚，扩大投资拉动增长。坚持把投资作为稳增长、调结构的第一动力，打好项目攻坚战，力争固定资产投资达到700亿元。加强项目谋划储备。围绕资源优势、区位特色、产业基础和政策机遇，深度谋划项目，储备具有全局性、基础性、战略性亿元以上大项目不少于300个。推进项目招商洽谈。抓住“民企吉林行活动”、第十届东博会等契机，深入开展“走出去”、“请进来”经贸交流活动，实施“五区布点、四步招商”方式，力争招商引资到位资金930亿元，增长15%。狠抓项目开工建设。实施亿元以上项目200个，对已签约项目抓开工，对已开工项目抓投产，对已运行项目抓达效，在项目建设数量、质量、规模和效益上实现新突破。严格项目管理考核。落实重大项目旬报告、月调度、季通报制度，建立项目谋划、洽谈、建设的进程档案。完善项目考核机制，突出项目落地、投产达效考核。

　　（二）突出提速增量，推进工业壮大跃升。围绕发展生态工业，应对经济下行，把稳工业作为稳增长的重要支撑，在提速增量上下功夫。力争规模以上工业增加值完成240亿元。兴产业。做好“有中生新”、“无中生有”两篇大文章，促进“五业振兴”。加快风电、光电产业开发，推进风电场建设，抓好洮南500千伏输变电工程，积极争取特高压项目，壮大金升镍业、富达棒材等高载能企业，有序推广清洁供暖、“柴改电”、光伏提水电灌农业试点，打造中国北方云计算中心；推进正邦、艾倍特等农畜产品加工项目；实施鑫红钻、通业等汽配企业技改项目；扶持中材等装备制造企业扩大生产；抓好四季盛宝异地搬迁等纺织服装项目。力争“五大产业”产值占规模以上工业总产值85%。强企业。围绕“三个三”目标任务，坚持引进增量与做大存量并举，滚动实施百户重点企业培育工程，搞好“双百”技术创新，抓好“两化”融合试点，推进306户规上企业发展升级。扶民营。落实发展民营经济、支持小微企业政策措施，加强小企业创业孵化基地建设，抓好“万名创业者、万名小老板”培训，在鼓励多生成、扶持快成长、推动早做强上下更大功夫。力争民营经济主营业务收入实现1185亿元，增长9%。促转型。加强开发区、工业园区平台建设，打造承接产业转移洼地，活化“飞地经济”模式，探索循环经济发展途径，争创国家级生态工业示范区。

　　（三）突出提质增效，促进农业现代化。大力发展生态农业，积极创建国家级现代农业示范区。做大规模。培育典型专业合作社100个，新发展家庭农场300个，推动土地流转125万亩，土地规模经营达到200万亩，农业综合机械化率达到73%以上。推进“六畜兴旺”，新（改、扩）建标准化规模养殖场（小区）200个，完善市、县、乡三级动物疫病防控体系。打响品牌。抓好500万亩优质特色农产品基地建设，无公害、绿色、有机农产品种植面积力争达到300万亩。加强国家级食源性食品安全生产基地建设，申请成立国家粗杂粮检疫检测中心。实施百个品牌推广工程，全力打造“有机米、健康豆、精品肉、生态鱼”品牌，让白城特色农产品走出去、卖个好价钱。完善设施。开工建设嫩江、洮儿河干流治理和月亮泡蓄滞洪区工程。加强小型农田水利建设，着力解决农田灌溉“最后一公里”问题。建设好高标准基本农田。抓好土地整理。推进节水增粮行动。实施好“粮安工程”，大力推动科学储粮仓建设。强化支撑。推广机械深松、保护性耕作等重大农业技术。创新农村金融服务方式。加强人工影响天气工作。推动惠农信息“进村入户”。实施百名农村致富能人培养工程。

　　（四）突出转型升级，加快服务业发展。着重发展新兴业态，改造传统服务业，实现量增质优，提升对经济增长贡献率。打造现代物流。重点推进各类物流园区建设，抓好国家外贸出口转型示范基地建设，提升德泰绿豆等省级出口基地功能。培育电子商务。发展互联网经济，深化与阿里巴巴战略合作，建设“特色中国?白城馆”，抓好“千县万村”计划试点，力争发展100个淘宝村镇、电商村镇。加快建设电子商务产业园。创建全国第二批信息消费试点城市。发展生态旅游。围绕“一线八点”旅游线路、“三大旅游品牌”，加快建设向海、莫莫格、嫩江湾、查干浩特等景区，启动建设军事文化产业园、包拉温都生态农场，争创国家级生态旅游示范区。力争实现旅游业收入46亿元，增长20%。创新金融服务。抓好农村金融综合改革试验，建立10个村级金融合作组织，推进信用联社改制；建立农村产权交易中心，组建市级物权融资公司；加大对农业产业化、农村基础设施、盐碱地改造等项目的信贷投放力度，推进土地收益保证贷款业务；县（市）村镇银行力争实现全覆盖。支持中小企业开展资本市场融资。开展“金融诚信年”活动。合理引导住房消费，推动房地产业健康发展。

　　（五）突出功能拓展，推进城镇化建设。围绕新型城镇化的要求，探索走出符合实际、特色鲜明、持续发展的白城城镇化之路。提升中心城市功能。加快生态新区核心区建设，抓好道路、管网、绿化等工程，推进碧桂园城市综合体、红星美凯龙家居卖场、深国投商业综合体等项目，抓好中绿农产品电子交易中心建设，启动建设文化体育公园。实施老城区改造提升工程，新建改造街路19条，改造供水管网24.6公里、排水（污水）管网33公里、供热管网10公里，铺设天然气管网10公里，搞好东湖泄水区生态恢复，改造污水处理厂，建设林业科普公园。加强县域城镇建设。突出抓好镇赉、洮南、通榆、大安等城市水系建设。推进林海、安广、兴隆山、黑水、坦途等小城镇试点。争取万宝镇独立工矿区纳入国家试点。深入实施新农村“富民强村固基”工程，加强新型农村社区建设。强化城市综合整治。开展“城市文明管理创建年”活动。加强城乡结合部、城中村综合整治。开展拆违打非、马路市场、交通秩序等专项治理。完善小区物业管理、市容保洁长效机制，加强社区网格化管理。推进“数字城市”建设。加快推进互联互通。白城机场力争竣工试航。抓好白阿、长白铁路提速改造和长西铁路新建项目，做好平齐、通让铁路电气化改造前期。推进嫩丹高速、嫩江大桥建设。启动国道302白城绕越线项目，新改建农村公路200公里。力争长庆街公铁立交桥竣工、丽江路公铁立交桥开工。实施好“宽带吉林”工程，提高村宽带接入覆盖面。

　　（六）突出瓶颈破解，推动重点领域改革。按照中央、省委和市委的部署，突出吉林西部生态经济区综合配套改革重点，推进全面深化改革。抓好经济领域改革。研究制定金融支持、财政投入等政策，培育壮大新型生产经营主体。做好农村集体土地承包经营权确权登记，建立农村土地承包经营权流转市场。加强农田水利设施产权制度改革。结合电力市场化改革，创新交易办法。推进工商登记制度改革，争取全程电子化工作试点。开展不动产统一登记工作。深化预算和国有资产管理改革。抓好行政领域改革。加大简政放权力度，完善事中事后监管。推进政府部门权力清单工作。加强政务大厅建设，开展重点投资项目并联审批。推进城市管理行政执法体制改革。抓好社会领域改革。完善科技成果转化应用的评价和奖励制度体系，加快产学研结合。整合教育资源，推进职业教育办学模式改革，探索人才培养新途径。启动现代公共文化服务体系改革试点。深化县级公立医院改革，逐步扩大覆盖面。创新户籍、社保、公共服务制度。抓好生态领域改革。落实最严格的水资源、耕地、湿地等保护制度，完善生态保护预警、治理修复、规划环评、执法监督等制度。开展资源有偿使用和生态补偿改革试点。探索财政投入、政策引导、社会参与的生态治理修复机制。

　　（七）突出保护治理，切实改善生态环境。围绕建设吉林西部生态屏障，创建国家生态保护与建设示范区。推进河湖连通。积极申请国家立项，加快单项工程审批和实施，力争新连泡塘13个。制定发展绿色农业、生态旅游、水产养殖、地产开发等规划，打造河湖连通产业链条。启动水生态文明试点城市一期工程。推进造林绿化。争取国家三北五期、新一轮退耕还林等项目，造林35万亩，圆满完成“三年再造百万林，建设西部大屏障”任务。推进草原治理。争取国家退牧还草和退耕还草试点，重点实施草原恢复治理项目，形成西部优质牧草产业链。推进湿地修复。实施生态补水工程，严格控制地下水开采。加快实施向海核心区生态移民工程。争取国家退耕还湿试点。加强环境保护。实施“蓝天工程”，启动国电龙华热电厂脱硝工程，淘汰市区30%燃煤小锅炉，抓好大气污染防治。实施“清水工程”，推进松花江流域水污染防治项目建设，严格控制污染物排放。

　　（八）突出民生福祉，统筹发展社会事业。坚持抓实、抓细、抓常，继续实施保基本兜底线工程，着力保障改善民生。扎实办好民生实事。一是推进创业带动就业，培育创业促就业基地20个，推动就业困难人员就业4000人，农村劳动力转移就业35万人次。二是提高社会保障水平，城乡居民基本医疗保险人均补助标准由320元提高到380元。提高城乡居民和企业退休人员养老保险待遇标准。三是强化住房保障，抓好新城家园棚户区安置房续建及配套工程，组织好房屋征收。建设廉租房5.5万平方米。“暖房子”改造50万平方米。改造农村危房1.3万户。四是改善供水条件，新建改造市区二次供水泵站10座，解决1.3万户居民用水难题。实施农村饮水安全工程，解决2万农村人口饮水安全。五是实施公交便民工程，新建候车亭10个、站牌274个，更新公交车70辆，建成停靠站2处。六是推进文化惠民工程，建立流动图书站，完成送戏下乡400场、送电影下乡1万场，建设城乡健身路径60个，新建白城速滑馆。七是完善社区养老服务，启动居家养老示范中心，建设"12349"社区居家养老服务信息平台。八是推进城乡教育普惠，新建农村标准化学校6所，维修改造校舍1.5万平方米。九是加强特困群体救助，提高城乡最低生活保障标准，分别达到上年收入的20%、25%。十是改善农村人居环境，抓好农村环境清洁、人畜分离等“六大工程”。集中力量扶贫攻坚。坚持以百个扶贫项目攻坚工程为载体，开展精准扶贫，抓好40个村整村推进，完成6.5万贫困人口脱贫。着力发展社会事业。继续实施百项实用技术对接工程，完成对接项目25项。抓好学前教育，推进义务教育均衡发展，统筹发展职业教育。深入搞好“歌舞鹤乡?放飞梦想”公共文化示范项目。加强文化产业园区、文化商业街、查干浩特体育公园建设。加快建设市中医院、卫生急救中心、残疾人康复中心、妇幼保健院。抓好人口和计划生育。加强国防教育，促进军民融合发展，努力建设“全国双拥模范城”。启动第三轮修志工作。开展地名普查。做好物价稳定工作。保障妇女儿童权益，发展老龄、慈善事业，推进民族宗教、外事侨务、人防、档案等工作。

　　（九）突出安全稳定，创新社会治理机制。加强大安全建设，深入开展“严查彻改去隐患、维稳防突保平安”活动。强化安全生产。全面开展“四化融合”、“三位一体”安全监管防控体系建设，实现“五个全覆盖”，坚决遏制重特大事故发生。强化依法处访。大力推行阳光信访、逐级走访、诉访分离。完善信访与仲裁、司法等渠道的衔接机制，妥善解决好群众信访问题。强化社会治安。深入推进“平安白城”建设，围绕维稳处突、侦查破案、治安防控、社会治理，提升打防管控和服务能力。加强网络安全管理。强化食药监管。深入实施食品药品安全放心工程，开展专项整治活动，确保群众饮食用药安全。强化应急管理。加强安全教育、培训和演练，完善公共安全、地质气象、防震减灾等预警体系，健全军警民应急救援机制，提高突发公共事件处置能力。抓好抗旱防洪、森林草原防火等工作。

　　（十）突出法治规范，加强政府自身建设。落实全面推进依法治市实施意见，坚持依法行政，加快建设法治政府。提升法治理念。推行政府常务会议和部门会议学法制度，办好科学行政讲堂，增强运用法治思维和方式推动工作的能力。深入推进“六五”普法。坚持依法办事。推进事权规范化、法定化，建立重大决策合法性审查机制，对重大决策建立终身责任追究制度。完善政府法律顾问制度，坚持3个联席会议制度。完善监督机制。自觉接受人大法律监督、工作监督和政协民主监督，认真办理人大代表建议和政协提案。完善政府内部层级监督和专门监督，加强审计监督，主动接受社会监督。推进政务公开。加强财政预算、公共资源配置、重大项目建设、社会公益事业等重点领域信息公开。抓好信息公开网络建设，建立社区、行政村信息公开平台。强化从严治政。巩固党的群众路线教育实践活动成果，持之以恒转作风。坚持从严管理干部，突出“管早、管小、管勤、管狠”。强化“三公”经费管理。完善绩效评价指标体系，健全抓落实工作机制。加强廉政风险防控机制建设，严肃查处违法违纪案件，以为民务实清廉的形象取信于民。

　　各位代表！

　　白城新一轮振兴发展正处在转型升级的重要关口，面对新机遇、新常态、新征程，让我们在省委、省政府和市委的正确领导下，在市人大、市政协的大力支持下，万众一心，开拓进取，奋发图强，共创白城美好的明天！
